# Effects of Tobacco Smoking

**Published:** 1863-10

**Authors:** 


### Selections.

Effects of Tobacco Smoking.—The effects of tobacco on the system has been a subject of discussion in the newspapers and medical and other scientific journals for some time past. B. W. Richardson, who certainly writes as though he was himself fond of the luxury of a smoke, comes to the following conclusions on the subject. They are published in the *London Lancet.*

"1. The effects that result from smoking are due to different agents imbibed by the smoker, viz., carbonic acid, ammonia, nicotine, a volatile empyreumatic substance, and a bitter extract. The more common effects are traceable to the carbonic acid and ammonia; the rarer and more severe to the nicotine, the empyreumatic substance, and the extract.

"2. The effects produced are very transitory, the poison finding a ready exit from the body.

"3. All the evils of smoking are functional in character; and no confirmed smoker can ever be said, so long as he indulges in the habit, to be well. But it does not follow that he is becoming the subject of organic and fatal disease because he smokes!

"4. Smoking produces disturbances in the blood, of the stomach, of the heart, of the organs of sense, of the brain, of the nervous filaments and sympathetic or organic nerves, of the mucous membrane of the mouth, and of the bronchial surface of the lungs.

"5. The statements to the effect that tobacco-smoke causes specific diseases—such as insanity, epilepsy, St. Vitus' dance, apoplexy, organic disease of the heart, cancer, and consumption—have been made without any sufficient evidence or reference to facts. All such statements are devoid of truth, and can never accomplish the object which those who propose them have in view.

"6. As the human body is maintained alive and in full vigor by its capacity within certain well-defined limits to absorb and apply oxygen, as the process of oxydation is most *active and most required* in those periods of life when the structures of the body are attaining their full development, and as tobacco-smoke possesses the power of arresting such oxydation, the habit of smoking is deleterious to the young.

"7. In the main, smoking is a luxury which any nation of natural habits would be better without. The luxury is not directly fatal to life, but its use conveys to the mind of the man who looks upon it calmly the unmistakable idea of physical detriment.

"8. But as a luxury tending to this condition, it is probably one of the least hurtful of luxuries. It is on this ground, in fact, that tobacco holds so firm a position; that of nearly every luxury it is the least injurious. It is innocuous as compared with alcohol; it does infinitely less harm than sugar (?); it is in no sense worse than tea; and, by the side of high living, altogether it contrasts most favorably. It is most antidotal to gluttony.

"9. Tobacco may also be considered, in certain cases, as a remedy for evils that lie deeper than its own, and as such a remedy it will persist in holding its place until those evils be removed."

On the above the *Dublin Medical Press* remarks as follows:

"The reader of the above will at once perceive that Dr. Richardson's observations are as mildly condemnatory of the practice of smoking as they well can be; while he admits that 'no confirmed smoker can ever be said, so long as he indulges in the habit, to be well;' while he enunciates the fact that 'as tobacco-smoking possesses the power of arresting oxydation, the habit of smoking is deleterious to the young,' yet he winds up by saying that 'it is one of the least hurtful of luxuries;' he makes the extraordinary statement that 'it does infinitely less harm than sugar,' and is antidotal to gluttony.' So far we must admit that Dr. Richardson is a most competent and unprejudiced judge; but as his verdict fails to condemn the use of tobacco on account of its direct effects, we must only fall back, in the absence of further and and more decisive evidence, on the common sense view of the case. Let us suppose that it is an open question whether tobacco be hurtful or not, and we think we can show that smoking is a practice which even the satisfaction of enjoying a luxury can not justify. In the first place, we would ask is it not culpably foolish for the smoker to create an appetite which does not exist in nature? If it were possible for a person to exist without drink, would it not be clearly wrong for him to tie himself for the future to a want which he may not have the means of gratifying, and how much more is it objectionable to create a necessity which entails inconvenience and expense? The smoker will say, 'but the pipe is a luxury, and repays us for the inconvenience and expense;' to which we reply, 'it is a luxury, because you have made it one, and a necessity of your own creation. If you had not made the pipe your master, you would not have felt the necessity of the luxury. The man who never smoked feels no loss, because he knows not the appetite.' In the second place, public decorum and politeness are not compatible with the use ef tobacco, and it is on this ground that the habit was attacked by the *Times*. Perhaps a smoker amongst smokers commits no breach of manners, for the callous olfactory senses of his company experience no discomfort; still he should remember that a large section of the public, and more especially those to whom politeness is particularly due, the aged and the fair sex, regard the smell of tobacco as no better than that of assafœtida, and he should consider that to lungs unaccustomed to the fumes of the fragrant leaf, smoke is as distressing as sulphureous vapors. If young men remembered this, if they refrained from annoying and inconveniencing their neighbors, for the purpose of satisfying their own morbid appetites, people might say, 'let them poison themselves so long as we are none the worse.' But it is a patent fact, which calls for the loudest public complaint, that they do *not* refrain, but rather force their vitiated taste upon the public on every occasion. A lady or an old gentleman can not enter a railway carriage, or any place of open air, public resort, without having their senses offended and their clothes be scented with the odor of stale tobacco; nor is it considered unworthy of a gentleman in this fast age to enter a ball-room or theatre redolent of the filthy perfume which is inseparable from his after-dinner pipe. No one has the right to outrage good manners to satisfy his morbid taste, and smokers should not abuse the good humor of the public by obtruding their bad taste where it is out of place; but if we are to admit that the question of injury to health is still an open one, we must admit that the opponents of the pipe have a *prima facie* case of the most conclusive description in favor of their view. It is altogether impossible and contrary to every analogy that persons can imbibe even the minutest doses of a virulent poison, such as nicotine, without suffering injury. If the smokers have no more forcible argument than the pleasure of smoking to adduce, they must be content to be enrolled with the opium eaters, who are ready to urge the same argument. The relative difference between the smoker and opium eater is the quantity of poison imbibed, and we find it very difficult to believe that a habit which necessitates such a system can be regarded otherwise than as a degrading vice, and worthy of public condemnation."—*Med. & Surg. Reporter.*

The Modern Use of Tobacco.—A Dublin medical gentleman, at present in one of the midland counties, writes to say, that it appears to him that the evil effects of smoking tobacco are every day becoming more and more apparent among the working classes. A considerable number of laborers have, in the course of his inquiries, come under his observation, who complained of pain and distress in the left side of the chest, and for which, by the most careful exploration and examination into general symptoms, he was quite unable to account, except on the supposition that they were produced by excessive smoking. Our correspondent further remarks, that the working people have of late years, in that part of the country to which he alludes, not only become more addicted to smoking, but changed their usual practice of using the pipe in a manner which he believes much more conducive to constitutional derangement than their method of smoking some years since. Formerly a laborer working in a field found considerable difficulty in lighting his pipe, and was obliged to resort to all kinds of expedients to procure fire, such as touch-paper with flint and seel, endeavors to keep a coal alive, etc., all of which were attended with such uncertainty, or occupied so much time, that "the smoke," though so ardently coveted, was usually per force postponed to stated times, when the man indulged in a good pipe full and had done with it for some hours. The invention of the lucifer match, however, shortly followed by a large increase in wages to the workman, formed an important era in the history of smoking. Tobacco, though so expensive an article, became more easy of access, and the facility with which it can now be lighted out of doors threw the temptation of its *frequent* use in a manner absolutely irresistible before the workman, whose habit now is to smoke small quantities, perhaps every hour or half hour, so as to keep himself almost continually slightly under the influence of this fascinating narcotic. We have great pleasure in inserting the above remarks collected from our correspondent's letter. The subject is a very interesting and important one— *Editorial in Dublin Medical Press.*

